# Assessing the Perceptions and Practice of Self-Medication among Bangladeshi Undergraduate Pharmacy Students

**DOI:** 10.3390/pharmacy6010006

**Published:** 2018-01-15

**Authors:** Md. Omar Reza Seam, Rita Bhatta, Bijoy Laxmi Saha, Abhijit Das, Md. Monir Hossain, S. M. Naim Uddin, Palash Karmakar, M. Shahabuddin Kabir Choudhuri, Mohammad Mafruhi Sattar

**Affiliations:** 1 Department of Pharmacy, Noakhali Science and Technology University, Sonapur, Noakhali 3814, Bangladesh; omarrezaseam@gmail.com (M.O.R.S.); phar_rita@yahoo.com (R.B.); bijoylaxmi.saha@yahoo.com (B.L.S.); abhijitdas@nstu.edu.bd (A.D.); monirjupharmacy@gmail.com (M.M.H.); 2 Department of Pharmacy, University of Chittagong, Chittagong 4331, Bangladesh; pharma.naim@yahoo.com; 3 Department of Pharmacy, Jahangirnagar University, Savar, Dhaka 1342, Bangladesh; mskchoudhuri@juniv.edu (M.S.K.C.); mafruhi1968@yahoo.com (M.M.S.)

**Keywords:** self-medication, pharmacy students, awareness, perception, Bangladesh

## Abstract

**Objectives:** To evaluate the perceptions and extent of practicing self-medication among undergraduate pharmacy students. **Methods:** This cross-sectional, questionnaire-based study was conducted over a six month period (January to June 2016) among undergraduate pharmacy students in five reputable public universities of Bangladesh. It involved face-to-face interviews regarding self-medication of 250 respondents selected by simple random sampling. **Results:** Self-medication was reported by 88.0% of students. Antipyretics (58.40%) were mostly preferred for the treatment of fever and headaches. The major cause for self-medication was minor illness (59.60%, *p* = 0.73) while previous prescriptions were the main source of knowledge as well as the major factor (52.80%, *p* = 0.94) dominating the self-medication practice. The results also demonstrated 88.80% of students had previous knowledge on self-medication and 83.60% of students always checked the information on the label; mainly the expiry date before use (85.60%). A significant (*p* < 0.05) portion of the students (51% male and 43% female) perceived it was an acceptable practice as they considered self-medication to be a segment of self-care. Furthermore, students demonstrated differences in their response level towards the adverse effect of drugs, the health hazard by a higher dose of drug, a physician’s help in case of side effects, taking medicine without proper knowledge, and stopping selling medicine without prescription. **Conclusions:** Self-medication was commonly used among pharmacy students primarily for minor illnesses using over-the-counter medications. Although it is an inevitable practice for them it should be considered an important public health problem as this practice may increase the misuse or irrational use of medicines.

## 1. Introduction

Self-medication is a human trait in which an individual (or a member of the individuals’ family) selects and uses medicines or any other substances for the treatment of self-recognized or self-diagnosed physical or psychological ailments [1]. Conventionally it has been described as the intake of drugs, herbs or other home remedies on an individual’s own persuasion or taking the advice of another person without consulting a physician [2,3]. Thus it forms an integral part of patients’ self-care which in fact is the first choice and is one of the most crucial tools when an individual encounters common health problems that do not require a doctor’s visit [4,5]. Due to insufficient medical facilities, the free accessibility of over-the-counter (OTC) drugs in the local market and the impoverished national drug regulatory policy, it is now becoming a very common occurrence in numerous countries of the world. Other reasons for self-medication are the shortage of time to visit a physician, inability to get a quick appointment, mild illness, long distance of hospitals and clinics from home, and finally unaffordable doctor’s fees. Moreover, extraction of much information from online sources, magazines or periodicals makes people courageous about treating their own illness [6]. However, people are endangering their lives by practicing self-medication as it can lead to habituation, lethal allergic reactions, under dosage of medication which may not alleviate the symptom, and also over dosage that can cause collateral injury to different organs [7]. 

The substances which are most extensively self-medicated are OTC drugs and dietary supplements. Besides analgesics, antimalarials, antibiotics, and cold syrups are intermittently used for self-administration [8]. Sometimes some psychoactive drugs like recreational drugs, alcohol, and comfort foods are self-medicated to alleviate the symptoms of mental distress, stress, and anxiety [9]. The practice of self-medication has become very familiar throughout the world [10,11,12,13] with a high prevalence rate in developing countries [14,15]. Some studies have found that the amplitude of self-prescribing rate with antibiotics in Asia is 4–7.5% which is higher that of 3% in northern Europe [16]. Although self-medication, when practiced precisely can save time and is also cost effective to the patients where professional care is relatively expensive and not readily available, there are several critical health hazard issues that should be considered before endorsing the potential benefits of self-medication [7,17,18]. Sometimes it may lead to wastage of resources, boost up resistance to pathogens, and cause severe health problems, including adverse drug reaction, addiction, and ultimately death [7]. 

There are no examples of data relating health hazards and health care utilization including the practice of self-medication among young adults, but it is expected that they are highly motivated towards self-administration of drugs by the internet and media [19]. A study carried out by the university students of Karachi, Pakistan demonstrated that the propensity of self-prescribing of medications among medical students was 77.7%, and was 83.3% for non-medical students [20]. So the study on self-practice of medications among university pharmacy undergraduates is imperative as they are that segment of the population who are well educated and have access to all the information regarding their health. Moreover study on the tendency of self-medication practice among pharmacy undergraduates is essential as they are the oncoming drug prescribers and health educationalist [5].

Over-the-counter (OTC) drugs are the only drugs which can be self-prescribed and sold in convenience stores, grocery stores, and health shops without prescription as they are less hazardous [21]. In Bangladesh paracetamol, ORS saline, metronidazole, ranitidine, omeprazole, aspirin, and diaclofenac sodium etc. are accepted to be sold as OTC drugs. However, due to immoral drug sellers and improper regulation, 90% of stocked drugs are sold without any prescription and therefore the phenomenon of self-medication is a common topic in our country [21]. Besides there have been very confined researches conducted regarding the impact of self-medication practice among university pharmacy undergraduates [15]. 

Considering all this evidence, this research work focuses on assessing the perceptions about self-medication practice among the pharmacy students of Bangladesh. The study also compares the attitudes toward and the extent of practicing self-care between male and female students, as well as the year in pharmacy school. 

## 2. Methods

### 2.1. Study Design

This population-based cross-sectional study was carried out to investigate the knowledge, attitudes and practice of self-medication among the undergraduate pharmacy students of Bangladesh from January to June, 2016. The study was conducted by using both qualitative and quantitative data.

### 2.2. Study Area

To conduct the study, the pharmacy department of five public universities of Bangladesh namely Dhaka University, Jahangirnagar University, Chittagong University, Comilla University, and Noakhali Science and Technology University were chosen as the study area. The above mentioned universities were chosen by the field investigators based on the availability and accessibility of the participants. Time and distance were also the key factors for the selection of the study areas.

### 2.3. Study Participants

The study included 250 undergraduate students (131 male and 119 female) enrolled in a Bachelor of Pharmacy program who understood English, aged between 18–25 years and were permanent residents of Bangladesh, with different socioeconomic backgrounds from five different public universities. Both residential and nonresidential students were selected randomly, i.e., we did not consider whether the students were resident or non-resident in different halls of the university. Before data collection each and every participant was clearly informed about the purpose of the study and a written consent was taken from each of the respondents. 

### 2.4. Participants and Eligibility Criteria

This study included only those respondents who were easily available for data collection and interested to provide information willingly. Those who did not feel comfortable to give information were excluded from the study.

### 2.5. Sampling and Sample Size

A simple random sampling technique was used for the selection of study participants. The sample size was calculated assuming that 50% of the undergraduate pharmacy students had a tendency of self-medication practice with 5% margin of error and 95% confidence interval. The sample size was calculated to be 232. However, to ensure more representative data, we selected a larger sample size of 250 for this study.

### 2.6. Data Collection

The procedure of data collection was segmented into three steps. The first step was to fill out the questionnaire including socio-demographic information by the study subjects. The second step was to discuss the study protocol, and the final step was to cross-check the questionnaires filled by the respondents. The questionnaire was adopted from a formerly published study which was developed, standardized, and previously used by Kumar et al. [4] for undergraduate pharmacy students. The questionnaire was divided into four segments and consisted of 16 close-ended and 11 open-ended questions. Section 1 contained questions related to socio-demographic information of the respondents. Section 2 included questions about the practice of self-medication by the respondents. Section 3 was concerned with the knowledge and awareness related questions of the respondents while Section 4 was on questions related to the perception of the respondents regarding self-medication practice. 

The questionnaire was distributed to the selected student together with a written consent form that explained the purpose of the research and assured them of their confidentiality. The interviews lasted for 15 min and included a range of questions about self-medication along with an explanation about self-medication, its main principles, as well as evidence based and practical demonstration. The questionnaire was constructed in English and translated to Bengali by the interviewers to make the questions easily understandable to the participants during the interview. They were asked to complete the questionnaire immediately. The authors were present on hand to answer questions or clarify any doubts that they might have. 

### 2.7. Statistical Analysis

Data analyses were conducted using SPSS software version 20.0 (SPSS Inc., Chicago, IL, USA). Descriptive statistics was used for the calculation of proportions. The Chi-square test was performed to measure the association between the demographic characteristics and responses to understanding, perceptions and self-use of medication. The *p* values were calculated by the Chi-square test. An alpha level of 0.05 or less was considered significant. The Microsoft excel program was used for data analysis and for chart, graph, and diagram preparation.

## 3. Results

### 3.1. Demographic Characteristics

Table 1 shows that 52.40% of the students were male while female students comprised 47.60%. Among all the pharmacy students 26% was from the 1st year, 26.00% from the 2nd year, 26.00% from the 3rd year, and 22.00% from the 4th year. The lowest number of students was in the age group <18 years with a percentage of 5.20% while the highest number of students was in the 21–25 years age group with a percentage of 63.60%. Another 31.20% of students was in the age group 18–20 years. Among the respondents 80.40% of respondents was from an urban area and 19.60% was from a rural area.

### 3.2. Practice of Self-Medication

Table 2 describes the practice of self-medication by pharmacy students (from 1st year to 4th year) from five reputable public universities of Bangladesh. From the table we get a clear scenario that the majority of students reported practicing self-medication (88.0%). The practice of self-medication among both male and female students (45.20% and 42.80% respectively) was almost similar with no significant difference (*p* > 0.05). 

### 3.3. Purpose of Self-Medication Practice

Table 3 demonstrates that the use of self-medication practice for different complications differed insignificantly (*p* > 0.05) between male and female students. It can also be seen from the table that students used self-medication for headache (71.20%); cough, cold/flu (61.20%); diarrhea (47.60%); pain (42.80%); stomach ache (32.80%); vomiting (32.00%); rash/allergies (23.60%), and skin problems (16.40%) respectively. Here the percentage of using self-medication for fever was highest and the value was least for ear problems. 

The purpose of self-practicing of medications by the students was categorized into seven broad categories (Table 3). The majority of the students (59.60%) used self-medication because they thought that they did not need to see a doctor for a minor illness. Easy availability of medicines (32.20%) and emergency use (32.00%) were the secondary reasons for practicing self-medication. Meanwhile quick relief (30.80%), lack of time to consult doctor (25.60%), sufficient pharmacological knowledge (19.20%), and cost effectiveness (12.80%) were other reasons behind self-medication of drugs. The table shows some high values on the purpose of using self-medication, but none of the values was significant (*p* > 0.05).

Different categories of medicine which were self-medicated by the students for the treatment of ailments are listed in Table 3. From the table it is clear that the use of different classes of drugs showed no difference in use between males and females. Among the drugs the rate of antipyretic consumption was highest (58.40%) by the students whereas analgesics (49.20%); anti-diarrheal (39.20%); antacid (38.80%); vitamins (31.60%); anti-allergic (29.20%); cosmetics (22.00%), and anti-diabetics (15.60%) were also used by them. It was found that among different types of drugs students used ophthalmic preparations the least with a percentage of 1.60%.

### 3.4. Sources of Information on Self-Medication Practice

**Figure 1:**
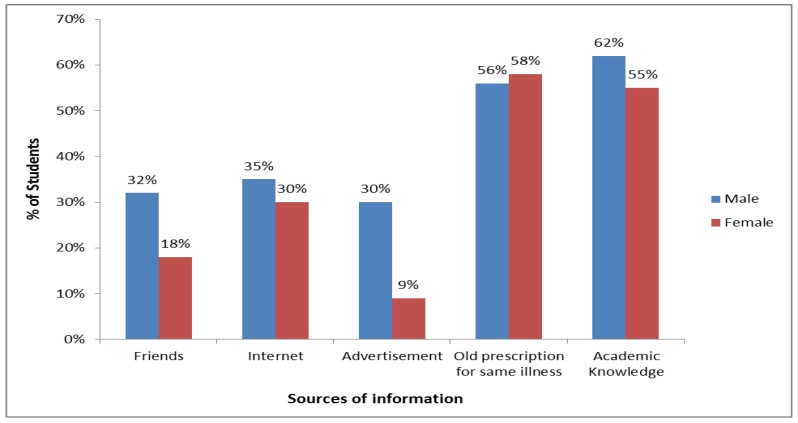
Sources of information on self-medicated drugs. Data is represented as percentage (%) for the two groups.

Figure 1 represents the pharmacy students’ (1st to 4th year) source of information on the medications self-prescribed by them. From the diagram we get a clear picture that the majority of the male and female students considered an old prescription for common illness (56% and 58% respectively) and academic knowledge (62% and 55% respectively) as their primary sources of information on self-medicated drugs. It was an interesting finding that male students gathered information about medicines from friends (32%), internet (35%), and advertisement (30%) and this rate was higher in comparison to female students.

### 3.5. Factors Influencing Self-Medication Practice

Table 4 represents a pharmacy student’s opinion on the factors (predefined categories from the survey) which influence the practice of self-medication. A major portion of the students (52.80%) said that they had self-prescribed a drug based on the previous doctor’s prescription for the same disease. Another 50.80% students had previous experience of practicing self-medication while the opinions of family members (45.20%) and of friends (15.20%) were also influential factors for self-medication practice by the students. Advertisement (6.00%) and recommendation by local people (5.60%) were the least influential factors for self-prescription of medications. 

### 3.6. Knowledge on Self-Medication Practice

Table 5 is concerned with the information relating to pharmacy students’ perceived knowledge on self-medication practice. The table illustrates that most of the students who had self-prescribed medication for different diseases knew about self-medication (88.80%) previously. Among the students 83.6% had a tendency to check the package insert while 85.6% students had a sound attitude towards checking the expiry date of the drug before using it. Furthermore 67.6% students had concerns about the importance of completing the course of the drug. All the results differed significantly (*p* < 0.05) when compared between male and female students. 

### 3.7. Attitude towards Self-Medication

**Figure 2:**
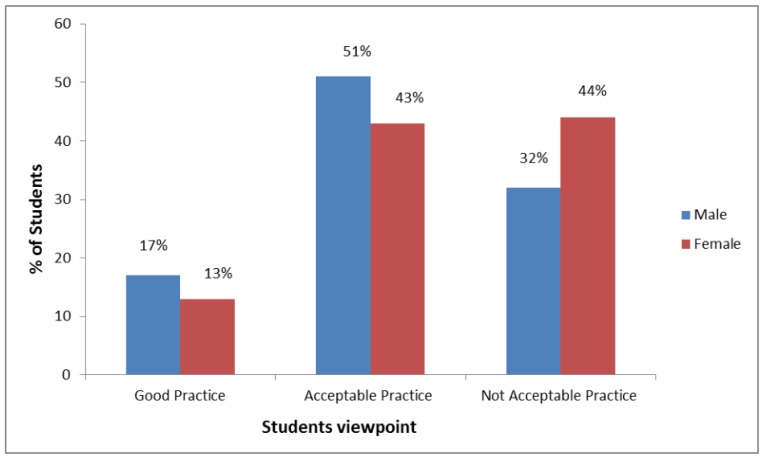
Attitude of the students towards self-medication. Data is represented as percentage (%) for the two groups.

Figure 2 represents the pharmacy students’ (1st to 4th year) approach on self-prescription of medication for self-healthcare. From the figure it is clear that the students’ concept about self-medication was classified into three categories namely good practice, acceptable practice, and not acceptable practice. Both male (51%) and female (43%) considered self-medication an acceptable practice while another 17% male and 13% female students considered self-practice of medication was good. Moreover the diagram also shows that 32% male and 44% female students thought self-medication was not an acceptable practice. 

Table 6 represents the students’ attitudes on self-medication among 1st to 4th year pharmacy students of five reputed public universities. The answer level is given as “yes” and “no”. The majority of students had a positive attitude on self-medication. A very negligible number of students had a negative attitude.

The tabulated results show differences in response level of the students to some survey items such as self-medication is a part of self-care; the idea about drugs side effects; health hazard on increased dose of a drug; physician’s help in case of side effects; taking medicine without proper knowledge, and stopping selling medicine without prescription. The observed answers were significant (*p* < 0.05) in the case of concern about the impact of increased dose of drug, for no need of drug treatment in mild medical cases, for overlooking physicians and not selling drugs without prescription, while for other modalities the results were not significant (*p* > 0.05).

## 4. Discussion

People have always been very cautious about their personal health status and for this they have used self-medication, a feature of healthcare, from ancient times. Although self-medication has many pros and cons it depends on who uses it and how it is used for self-treatment [4]. We focused on pharmacy students because they have adequate knowledge of medicine in theory and are more cautious about the safety of drugs which is lacking in other student groups or in the general population. Thus a pharmacy student’s view on the self-medication practice can be considered as a major factor to judge the characteristics of their future prescription pattern. 

Students of Bangladesh frequently use self-medication and gender difference has not been shown to have any influence on the practice of self-medication. The reason behind insignificant gender differences in the overall exercise of self-medication may be the study format that allowed the respondents to select drugs by themselves [5]. In our study we found that about 88.0% of the students self-practice different types of medication. A similar type of study was conducted by Kumar et al. [4] in coastal south India and signified that the amplitude of self-medication practice was 78.90% among medical students. Other similar studies also demonstrated the prevalence rate of self-medication ranged between 57.1% and 92% among the medicals students in India [22]. Several research works carried out in other developing countries revealed that the prevalence of self-medication was 38.5% and 43.2% among medical, pharmacy, and health science students in Ethiopia [22,23], 51% among citizens in Slovenia [24], 55.3% and 55% among medical students in Pakistan [25] and Egypt [26] respectively, 56.9% among medical undergraduate students in Nigeria [27], and 80.9% among female university students in Malaysia [28]. The major influential reason behind the higher propensity of self-medication might be the unregulated easy availability of all categories of medicine without prescription.

Similar to some previously published articles [22,29,30,31], headache, common cold, fever, pain, and vomiting were the most common symptoms for self-administration of medications mentioned by the respondents. It was quoted in our research report that the most common cause for self-treatment with drugs was the insignificance of the illness which did not require a doctor’s visit. Similar outcomes were reported by the study conducted in India [7,32]. This type of attitude of the respondents may be attributed to a disregard and absence of consciousness about the advancement of diseases. Sometimes the people who practice medication for self-treatment may suffer from a serious illness as the symptoms of many diseases are primarily mild but wrong diagnosis and treatment may promote serious health hazards. However, in agreement with other studies, easy availability of medicines [4,6], quick relief [16], and time saving [33] were found to be the other causatives for preferring self-medication practice. 

As stated earlier, antipyretics, analgesics, antacids, and antidiarrheal drugs were the most common classes of drugs self-prescribed for treatment by almost all of the respondents in our study. Almost identical observations were found in the studies conducted in India [4,34], Pakistan [32], Iran [35], and Ethiopia [8] where these common classes of drugs were frequently used by medical students. Meanwhile, the use of antibiotics was different to that of analgesics and antipyretics. This tendency is because of the knowledge of pharmacy graduates on the resistance and side effects of antibiotics. It is well known that proper medicinal knowledge can promote a good prescribing pattern of pharmacists. However, at the same time inappropriate or irrational use of these drugs can lead to various hazardous effects including the reduction in the capability of microbial flora to resist detrimental microorganisms, the development of multidrug resistance, addiction, toxicity, and other related syndromes [32]. Therefore, such kind of practice should be discouraged.

Our study found that the key factor for self-medication practice by the participants was their adequate pharmacological knowledge which they had gathered from their academic courses. These findings are similar to those from studies conducted in Nepal [7], India [4,33], Malaysia [36], Ethiopia [8], and Pakistan [32]. The second major source of information on self-prescribed drugs was from previous prescriptions for the same illness and this result was analogous to the findings of the study conducted in India [4,33]. Further, other researches conveyed in India [34] and Ethiopia [8] reported the internet as another common source of knowledge on self-prescribed medicines which was the third common source of information in our study results. 

The fact that majority (52.80%) of the respondents gathered information about self-medication from the previously prescribed medicines of physicians was consistent with the research work conducted earlier [37]. However as the respondents were younger, they were also influenced by other sources like previous illness experiences, opinions of family members, friends and local people, and advertisement. This result resembles formerly conducted research findings [23,37]. All the students irrespective of the year of the study reported that they were completely aware of the treatment procedure using self-medication. They were also cautious about completing the dose of the medicine, checking the instructions given on the insert before using and also looking for the expiry date of the drug before using it. Less awareness was noted among 1st year students which was similar to the findings of Kalyan et al. [38] and Sontakke et al. [34] Non-inclusion of pharmacology as a subject in the 1st year curriculum could be the reason.

In this research work, about 73.2% of the respondents believed the practice of self-medication to be part of their own health-care and the proportion was higher than the reports from India [34], Ethiopia [22], and Pakistan [25]. Self-medication can only be considered a part of self-care if legitimate use of medicaments can be ensured. It may lead to accidental drug toxicity as there is always a risk of using expired drugs and also sharing with friends or taking medicines that have been actually prescribed for other problems [4,29,32,39].

## 5. Limitations

The study had some limitations as we faced some complications during the survey. First, we covered only five universities due to shortage of time for the research work. If we had conducted the study in more universities we would have got a more extensive scenario on the self-medication practice among the university students of Bangladesh. Second, many students were busy with their examinations, and lab work for training purposes; so collecting data from them was slightly difficult. Third, students of 1st year and 2nd year were less familiar with some terminologies and complication were created during the understanding of the questionnaire. They had to be given extra explanation. Finally, social desirability bias may have impacted the responses since the interviews were done in person Also the survey did not distinguish between the uses of OTC drugs in self-medication vs. prescription drugs such as antibiotics and may have resulted in confusion among respondents. The survey was not revalidated after translation and may have resulted in some changes in the survey items.

## 6. Conclusions

This descriptive study has demonstrated that self-practice of medication is very common among undergraduate pharmacy students of five renowned universities which was facilitated by the easy availability of drugs and information from previous prescriptions. The use of antibiotics, antidepressants, and sedative among a small segment of students without proper follow up or lab tests by healthcare providers may lead to serious health hazards, not only to the students themselves but also to those to whom they suggest the medication. Therefore, it is the sole responsibility of the health care professionals and drug regulatory authorities to ensure the safe use of drugs and control the exercise of self-administration of medications by describing the total impact of the drugs on the body to the students. As the study was confined to the pharmacy student population, further research is needed to test the prevalence of self-medication practices among the general population and how these differ by type of medication. Furthermore steps should be taken to monitor the drug selling system by stake holders especially of those drugs with potentially harmful effects.

## Figures and Tables

**Table 1:** Demographic characteristics of respondents.

Item	Sub Group	Number (n)	Percentage (%)
Sex	Male	131	52
	Female	119	48
Year of study	1st year	65	26
	2nd year	65	26
	3rd year	65	26
	4th year	55	22
Age group	<18 year	13	5
	18–20 year	78	31
	21–25 year	159	64
Area of residence	Urban	201	80
	Rural	49	20

**Table 2:** Practice of self-medication of the respondents.

Item	Response	Male		Female		Total (%)	*p*-Value
		n	%	n	%		
Practice of self-medication	Yes	113	45.20	107	42.80	220 (88.0)	0.44
	No	18	7.20	12	4.80	30 (12.0)	

n indicates the number of respondents. *p* value was determined using Chi-square Test. *p* < 0.05 was considered significant when compared between male and female groups.

**Table 3:** Purpose of practicing self-medication of the respondents.

Items	Male		Female		Total (%)	$x^{2}$ Value	*p* Value
	n	%	n	%			
**Indications for Using Self-Medication**							
Headache	89	35.60	89	35.60	178 (71.20)	1.59	0.66
Cough, Cold/Flu	75	30.00	78	31.20	153 (61.20)	1.41	0.72
Fever	98	39.20	93	37.20	191 (76.40)	1.62	0.67
Stomach ache	34	13.60	48	19.20	82 (32.80)	1.88	0.62
Diarrhea	66	26.40	53	21.20	119 (47.60)	2.76	0.43
Menstrual Symptoms	0	0	12	4.80	12 (4.80)	-	-
Rash/Allergies	26	10.40	33	13.20	59 (23.60)	2.46	0.49
Anxiety/Depression	7	2.80	10	4.00	17 (6.80)	3.29	0.45
Ear Problems	3	1.20	2	0.80	5 (2.00)	2.10	1.00
Vomiting	37	14.80	43	17.20	80 (32.00)	4.54	0.21
Eye infections	7	2.80	9	3.60	16 (6.40)	3.81	0.13
Skin problems	27	10.80	14	5.60	41 (16.40)	1.02	0.83
Tooth ache	17	6.80	11	4.40	28 (11.20)	0.41	1.00
Insomnia	3	1.20	3	1.20	6 (2.40)	3.13	1.00
Pain	51	20.40	56	22.40	107 (42.80)	5.20	0.15
**Reason for Self-Medication**							
Minor illness	72	28.80	77	30.80	149 (59.60)	1.35	0.73
Sufficient pharmacological knowledge	27	10.80	21	8.40	48 (19.20)	1.56	0.74
Quick relief	40	16.00	37	14.80	77 (30.80)	2.17	0.56
Lack of time to consult doctor	35	14.00	29	11.60	64 (25.60)	3.99	0.26
Cost effectiveness	22	8.80	10	4.00	32 (12.80)	1.28	0.86
Easy availability of medicine	52	20.80	36	14.40	88 (35.20)	3.02	0.39
Emergency use	34	13.60	46	18.40	80 (32.00)	2.19	0.53
**Type of Self-Prescribed Medicine**							
Analgesics	63	25.20	60	24.00	123 (49.20)	0.60	0.93
Antipyretics	44	17.60	53	21.20	146 (58.40)	2.44	0.48
Antidiarrheals	48	19.20	50	20.00	98 (39.20)	2.64	0.44
Antiemetics	13	5.20	20	8.00	33 (13.20)	4.30	0.22
Antibiotics	27	10.80	12	4.80	39 (15.60)	1.73	0.66
Antacids	71	28.40	75	30.00	97 (38.80)	3.58	0.31
Sedatives	11	4.40	12	4.80	23 (9.20)	4.38	0.20
Anti-allergic	36	14.40	37	14.80	73 (29.20)	3.15	0.38
Vitamins	40	16.00	39	15.60	79 (31.60)	7.62	0.05
Ophthalmic preparations	1	0.40	3	1.20	4 (1.60)	-	1.00
Cosmetic products	21	8.40	34	13.60	55 (22.00)	1.72	0.64

Data is represented both as number and percentage (%). n indicates the number of respondents. *p* values from Chi-square or Fisher Exact tests for comparisons between male and female groups.

**Table 4:** Influencing factors for the selection of medications for self-practice by the respondents.

Factors	Male		Female		Total (%)	$x^{2}$ Value	*p* Value
	n	%	n	%			
Opinion of family members	52	20.80	61	24.40	113 (45.20)	1.74	0.63
Opinion of friends	27	10.80	11	4.40	38 (15.20)	1.62	0.65
Recommendation by local people	10	4.00	4	1.60	14 (5.60)	1.14	0.57
Previous doctor’s prescription	58	23.20	74	29.60	132 (52.80)	0.40	0.94
Own experience	75	30.00	52	20.80	127 (50.80)	9.08	0.03 *
Advertisement	12	4.80	3	1.20	15 (6.00)	1.63	0.44

n indicates the number of respondents. *p* value was determined using Chi-square Test.* *p* <0.05 was considered significant when compared between male and female groups.

**Table 5:** Knowledge of the students on self-medication.

Modality	Male		Female		Total (%)	$x^{2}$ Value	*p* Value
	n	%	n	%			
Idea about self-medication	130	52.00	92	36.80	222 (88.80)	8.84	0.03 *
Knowledge about dose completion of self-prescribed medications	90	36.00	79	31.60	169 (67.60)	14.16	0.00 *
Checking of the insert	120	48.00	89	35.60	209 (83.60)	7.54	0.03 *
Checking of the expiry date before use	110	44.00	104	41.60	214 (85.60)	8.17	0.04 *

n indicates the number of respondents. *p* value was determined using Chi-square Test. * *p* < 0.05 was considered as significant male and female groups.

**Table 6:** Attitude of the students towards Self-medication.

Modality	Year of Study	Yes/No (n)	$x^{2}$ Value	*p* Value
Part of self-care	1st	44/21	3.27	0.12
	2nd	47/19		
	3rd	47/19		
	4th	45/10		
Advice of self-medication to your family and friends	1st	29/36	0.50	0.98
	2nd	27/38		
	3rd	26/39		
	4th	25/30		
Idea about which drugs have side effects	1st	37/28	6.35	0.22
	2nd	49/16		
	3rd	48/17		
	4th	37/18		
Concern that increasing drug dose can be health hazardous	1st	45/20	12.80	0.00 *
	2nd	56/9		
	3rd	59/6		
	4th	48/7		
In the case of side effects a physicians help must be needed	1st	59/6	3.29	0.66
	2nd	63/2		
	3rd	61/4		
	4th	49/6		
Mild medical problems do not need drug treatment	1st	21/44	9.25	0.02 *
	2nd	29/36		
	3rd	24/41		
	4th	32/23		
Physicians can be overlooked	1st	43/22	11.26	0.00 *
	2nd	40/25		
	3rd	40/25		
	4th	21/34		
Taking without proper knowledge is harmful	1st	54/11	4.09	0.16
	2nd	60/5		
	3rd	60/5		
	4th	50/5		
Stopping selling medicine without prescription	1st	56/9	6.01	0.03 *
	2nd	56/9		
	3rd	58/7		
	4th	54/1		

n indicates the number of respondents. *p* value was determined using Chi-square Test. * *p* < 0.05 was considered significant for male and female groups.
